# Exploring dermoscopic structures for melanoma lesions' classification

**DOI:** 10.3389/fdata.2024.1366312

**Published:** 2024-03-25

**Authors:** Fiza Saeed Malik, Muhammad Haroon Yousaf, Hassan Ahmed Sial, Serestina Viriri

**Affiliations:** ^1^Department of Computer Engineering, University of Engineering and Technology, Taxila, Pakistan; ^2^School of Computing, College of Science, Engineering and Technology, University of South Africa (UNISA), Pretoria, South Africa; ^3^Barcelona Institute of Global Health (ISGlobal), Barcelona, Spain; ^4^School of Mathematics, Statistics and Computer Science, University of KwaZulu-Natal, Durban, South Africa

**Keywords:** dermoscopic structures, melanoma, Vision Transformers, medical imaging, PH2, Derm7pt

## Abstract

**Background:**

Melanoma is one of the deadliest skin cancers that originate from melanocytes due to sun exposure, causing mutations. Early detection boosts the cure rate to 90%, but misclassification drops survival to 15–20%. Clinical variations challenge dermatologists in distinguishing benign nevi and melanomas. Current diagnostic methods, including visual analysis and dermoscopy, have limitations, emphasizing the need for Artificial Intelligence understanding in dermatology.

**Objectives:**

In this paper, we aim to explore dermoscopic structures for the classification of melanoma lesions. The training of AI models faces a challenge known as brittleness, where small changes in input images impact the classification. A study explored AI vulnerability in discerning melanoma from benign lesions using features of size, color, and shape. Tests with artificial and natural variations revealed a notable decline in accuracy, emphasizing the necessity for additional information, such as dermoscopic structures.

**Methodology:**

The study utilizes datasets with clinically marked dermoscopic images examined by expert clinicians. Transformers and CNN-based models are employed to classify these images based on dermoscopic structures. Classification results are validated using feature visualization. To assess model susceptibility to image variations, classifiers are evaluated on test sets with original, duplicated, and digitally modified images. Additionally, testing is done on ISIC 2016 images. The study focuses on three dermoscopic structures crucial for melanoma detection: Blue-white veil, dots/globules, and streaks.

**Results:**

In evaluating model performance, adding convolutions to Vision Transformers proves highly effective for achieving up to 98% accuracy. CNN architectures like VGG-16 and DenseNet-121 reach 50–60% accuracy, performing best with features other than dermoscopic structures. Vision Transformers without convolutions exhibit reduced accuracy on diverse test sets, revealing their brittleness. OpenAI Clip, a pre-trained model, consistently performs well across various test sets. To address brittleness, a mitigation method involving extensive data augmentation during training and 23 transformed duplicates during test time, sustains accuracy.

**Conclusions:**

This paper proposes a melanoma classification scheme utilizing three dermoscopic structures across Ph2 and Derm7pt datasets. The study addresses AI susceptibility to image variations. Despite a small dataset, future work suggests collecting more annotated datasets and automatic computation of dermoscopic structural features.

## 1 Introduction

Melanoma, cited as the most lethal form of skin cancer, originates in cells known as melanocytes, responsible for imparting color to our skin through the synthesis of the pigment melanin. Excessive exposure to ultraviolet radiation from the sun is the primary cause of the disease, leading to mutations in melanocytes and resulting in melanoma genesis. The incidence of this disease is on the rise annually in both males and females. As one of the most prevalent cancer types, the Cancer Society estimates that in the United States for the year 2023, approximately 97,610 individuals will be diagnosed with melanoma (60% males and 40% females), with an 8% mortality rate (6.7% in men and 3.3% in women) (Team TACSm and editorial content, 2023). Encouragingly, melanoma is curable in up to 90% of cases if detected at the earliest stage (El-Khatib et al., 2020). However, if left undetected or misclassified, the chances of survival plummet to only 15–20%. Globally, countries with a very high Human Development Index account for approximately 85.7% of melanoma-based skin cancers and 67.2% of melanoma-related deaths. Projections suggest that by 2040, new cases of melanoma will see an increase of over 50%.

The task of differentiating between typical benign nevi and advanced melanomas remains a challenging issue. As melanoma develops in melanocytes, the detection of these pigmented lesions poses a challenge for dermatologists due to clinical variations (Stiff et al., 2022). Clinical variation refers to differences in the type, frequency, and costs of medical care provided to patients, irrespective of their condition. Resolving the considerable clinical variability in this area requires focused study. Enhancing the quality and effectiveness of healthcare for all patients becomes possible by identifying the causes of clinically divergent conditions and taking steps to reduce them. Concerning melanoma, the standard method for examining lesions is a visual analysis by experts, who consider asymmetry, border, color, diameter, and evolution commonly known as the ABCDE method, a manual diagnostic approach. This method can be time-consuming and may lead to misdiagnosis due to the lack of experience and fatigue of dermatological specialists (Vestergaard et al., 2008).

Although biopsy is the conventional method for detecting cancer, it is essential to anticipate that biopsy provides information confined to scar tissue, covering only the site where the disease begins (Nelson et al., 2019). Without the use of epiluminescence microscopy, also known as dermoscopy, dermatologists have achieved 65–80% accuracy, facing difficulties due to cosmetically sensitive features of the face (Stiff et al., 2022). However, in most cases, the utilization of dermoscopic images has improved diagnostic accuracy by up to 84%, which falls short of desirable medical diagnostic standards (Ali and Deserno, 2012; Fabbrocini et al., 2011). Due to the challenges in diagnosis, dermatologists have found assistance in Computer-Aided Diagnosis (CAD) systems incorporating dermoscopic images, necessitating a basic understanding of AI (Artificial Intelligence) (Nelson et al., 2019). Computer-aided diagnosis (CAD) represents a type of medical technology using computers to aid healthcare professionals in diagnosing diseases. CAD systems employ algorithms to analyze medical imaging, such as X-rays, MRIs, and CT scans, for the detection of potential abnormalities. Overall, CAD-based systems have the potential to enhance the accuracy and effectiveness of diagnosis.

In modern image analysis, a drawback in training AI-based models arises from slight alterations in input images, such as scaling or rotation, which are commonly encountered based on the conditions of image acquisition. However, even these minor changes in input images can significantly impact the classification capabilities of the training models. This adverse effect on the training models is referred to as brittleness. It is important to note that brittleness differs from adversarial attacks, which are specifically crafted to deceive deep learning models; instead, it represents fluctuations occurring during image acquisition. This vulnerability of AI tools has been disclosed within the machine learning community (Azulay and Weiss, 2018; Engstrom et al., 2019). This uncertainty has a substantial effect on clinical routines; therefore, addressing it is crucial for the successful integration of AI diagnostic tools into everyday clinical practice. Color constancy, as a pre-processing step in dermatological image analysis, offers significant benefits by effectively compensating for color shift variations in AI frameworks. Numerous studies have demonstrated its efficacy in standardizing image illumination sources, addressing the impact of variable illumination conditions on skin lesion diagnosis (Barata et al., 2014; Salvi et al., 2022). By mitigating the effects of color variations during image acquisition, color constancy enhances the accuracy of computer-aided diagnosis systems. In Salvi et al. (2022), Dermatological Color Constancy Generative Adversarial Network (DermoCC-GAN) is proposed to standardize image illumination sources, surpassing existing color constancy algorithms. The GAN is trained to perform domain transfer from original to color-standardized images, demonstrating superior performance in dermatological image analysis compared to state-of-the-art algorithms. The approach achieves high accuracy in lesion classification (79.2%) and segmentation (dice score: 90.9%), with validation on external datasets. The study highlights the potential of the proposed strategy for broader applications beyond dermatology.

**Figure 1:**
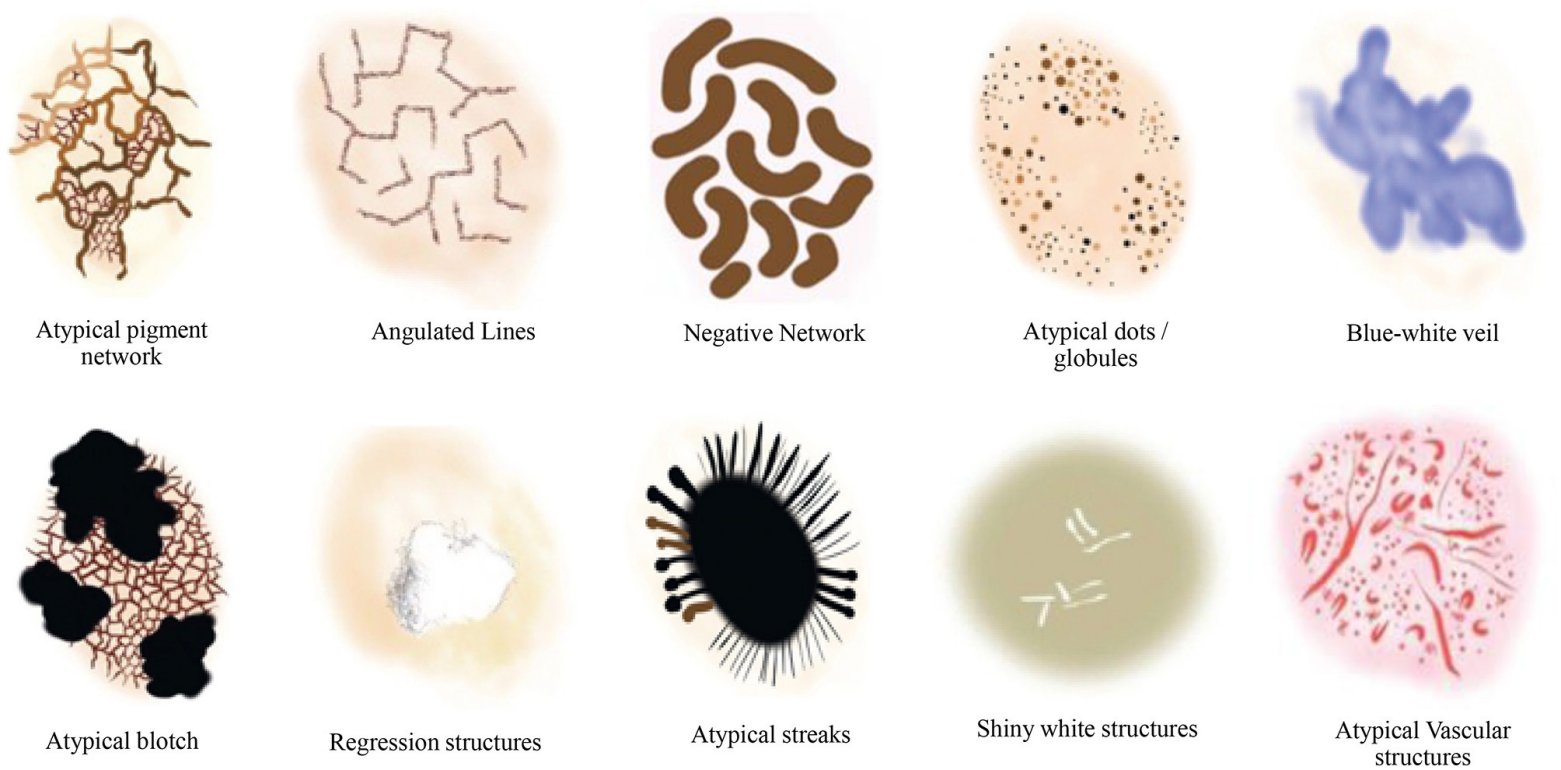
Literature survey for the presence of various dermoscopic structures for melanoma classification and recognition (Argenziano et al., 2003; Braun et al., 2005, 2002; Scope et al., 2006; Stricklin et al., 2011).

Additionally, the susceptibility of AI to image variations was investigated in Argenziano et al. (2003) by training deep learning-based models to discriminate between melanoma and benign lesions using typical features such as size, color, and shape. The testing set was divided into two categories: one with minor artificial changes, including rotation and zooming, and the other with minor natural variations, such as capturing images of the same lesion with changes in angles and lighting conditions, etc. The analysis revealed that artificial changes caused the outputted class to vary from melanoma to benign or vice versa, with a probability ranging from 3.5% to 12.2% (Argenziano et al., 2003). This significant reduction in classification accuracy with simple image transformations indicates the necessity of incorporating additional detailed information, such as dermoscopic structures. We propose a technique based on Transformer-based classification models that utilize dermoscopic structures as features. The Transformers-based model was trained with convolutions, and the results were compared with traditional CNN (Convolutional Neural Networks) based models employing dermoscopic structures as features. Various dermoscopic structures specific to melanoma classification are illustrated in Figure 1.

Our contributions are outlined as follows:

1. We conducted a comprehensive study on dermoscopic structures as features in melanocytic lesions.
2. We explored various approaches ranging from traditional Convolutional Neural Networks to Vision Transformers and OpenAI CLIP. Moreover, we enhanced the Vision Transformers by introducing convolutions for melanoma lesion classification.
3. We investigated the robustness of the models against image variations by assessing their performance on diverse versions of test sets.
4. We performed a comparative analysis of CNN-based models and Vision Transformers when utilizing dermoscopic structures as features, instead of the commonly employed features such as color, size, and shape of lesions. Additionally, we tested the highest accuracy model on ISIC 2016 images to detect the presence of dermoscopic structures.

The following sections of this paper are organized as follows. In Section 2, we delve into the related literature. Section 3 outlines the proposed technique, while Section 4 provides details on the datasets and evaluation matrices used to assess performance. We examine various models, including Vision Transformers, Clip, and CNN-based architectures like Vgg-16, DenseNet-121, and ResNet-50, along with the approach of training Transformers with convolutions using dermoscopic structures as features. The experimental setup is also thoroughly discussed. Subsequently, we compared the experimental results with existing work that relies on features other than dermoscopic structures. The paper concludes with Section 5, where the key findings are summarized.

## 2 Related work

To automate the skin cancer classification process, various artificial intelligence-based systems have been proposed, encompassing standard phases of pre-processing, feature extraction, segmentation, and classification. Many of these classification approaches relied solely on hand-crafted features, which, despite their deep understanding of biological patterns, exhibited limited capacity for generalizing to dermoscopic images (Xie et al., 2016; Barata et al., 2018). Moreover, the high correlation among lesions in terms of size, color, and shape, coupled with their substantial visual resemblance, resulted in degraded feature information (Celebi et al., 2007). This leads to the conclusion that hand-crafted features are unsuitable for classification purposes. Deep learning techniques offer the advantage of direct application to classification problems without the need for any pre-processing steps. In comparison to shallow networks, deep learning techniques prove more efficient in calculating features for lesion classification purposes. Esteva et al. (2017) introduced the first application of DCCNs (Deep Convolutional Neural Networks) to skin cancer classification, utilizing a pre-trained Inceptionv3 model on 129,450 clinical images to classify 2032 different diseases. A comparison with a board of 21 medical specialists was conducted, focusing on the classification of the two most common and deadliest types of skin cancers: malignant and nevus. Experts approved that their network exhibited high-performance lesion classification. Li and Shen (2018) proposed LICU (Lesion Index Calculation Unit), evaluated on the ISIC 2017 dataset, which filtered coarse classification by computing heat maps of outcomes from the FCRN (Fully Convolutional Residual Networks) model. This unit, for classification computation, predicted the contribution of each pixel from the segmented map.

The intrinsic self-attention ability of DCNNs was explored by Zhang J. et al. (2019). For the purpose of skin lesion classification, Attention Residual Learning (ARL) was implemented using CNNs, which featured multiple ARL blocks followed by global average pooling and classification layers. The classification performance was enhanced by employing a residual learning mechanism against each ARL block, generating attention maps at lower layers. Iqbal et al. (2021) designed a multi-class classification model for skin lesion assessment using DCNNs, evaluated on datasets of ISIC 2017, 2018, and 2019. Their model comprised multiple blocks with 68 convolutional layers to transmit feature information from the top to the bottom of the network. Similarly, Jinnai et al. (2020) employed Faster Region-Based Convolutional Neural Network (FRCNN) to classify melanoma using 5846 clinical images instead of dermoscopy. They prepared the training dataset by manually creating bounding boxes around the lesion areas; however, FRCNN outperformed ten certified dermatologists and ten trainee dermatologists by providing high accuracy.

A technique for improving performance metrics, including accuracy, AUC (Area Under the Curve), and others, using ensemble CNN models was studied by Barata et al. (2018). To conduct a three-class classification, the outputs from the classification layers of four distinct models: GoogleNet, AlexNet, ResNet, and VGG were fused. To further enhance classification performance, Yap et al. (2018) proposed a model incorporating various image modalities, including patients' metadata. On dermoscopic and macroscopic images, ResNet50 was applied differently, and the features were fused to predict classification. Using only macroscopy, their multimodal ResNet50 outperformed the basic model, showing an AUC of 0.866. Similarly, an ensemble model for multi-class classification, designed from EfficientNets, SENet, and ResNeXt WSL, was proposed by Gessert et al. (2019) on ISIC 2019 dataset. To handle multimodal input resolutions, a cropping strategy on input images was applied, and a loss-balancing approach was implemented for imbalanced datasets. Srinivasu et al. (2021) presented a DCNN based on MobileNetv2 and LSTM (Long Short-Term Memory), performing lesion classification on HAM10000 dataset. Compared to other CNN models, MobileNetv2 resulted in a reduced network size and low computational cost, providing compatibility with mobile devices. LSTM retained the timestamp information of features calculated by MobileNetv2, enhancing system accuracy by 85.34%. Conversely, ensemble-based deep learning models are computationally more expensive, requiring considerable training time. Their interpretation is challenging due to multiple layers of abstraction. Such models are sensitive to data quality and prone to degraded performance and overfitting in the presence of noisy or biased data and complex base models, respectively.

Ahmed et al. (2023) proposed a hybrid method integrating RetinaNet and MaskRCNN with a pyramid module for spatial feature compensation. Tested on Melanoma-ISIC-2018 and PH2 datasets, their approach demonstrates superior generalization, outperforming Encoder-Decoder, GAN, DCNN-SVM, EFCN, ECDNs, UNet, and Handcrafted methods by 7.7%, 12.9%, 11.4%, 14.4%, 14.9%, 18.6%, and 25.1%, respectively, in accuracy. With its promising accuracy, their method holds potential for future clinical application. Vocaturo et al. (2020) explored the application of multiple-instance learning (MIL) to discriminate melanoma from dysplastic nevi and further classified dysplastic nevi from common ones using the PH2 dataset among others. Specifically, they proposed using MIL with spherical separation surfaces, showing promising results. This suggested that MIL techniques could form the foundation for advanced tools in lesion detection.

Wang et al. (2021) proposed another method called STCN (Self-supervised Topology Clustering Network). This method contributes to classifying unlabeled data without the need for any prior class information. Depending on maximizing modularity, the clustering-based algorithm organizes the anonymous data into clusters. At different levels, STCN learns features such as illumination, background, and point of view. Several pre-trained models, including Xception, AlexNet, VGGNet, and ResNet, were studied using transfer learning, and their performance was compared in Kassani and Kassani (2019) and Jojoa Acosta et al. (2021). The hyperparameters were fine-tuned to enhance performance, and the fully connected layers were modified to use existing networks for skin lesion classification. For a deep understanding of using CNNs for skin cancer classification, systematic review articles (Haggenmüller et al., 2021; Höhn et al., 2021) can be referred to. The possible solutions for automatic skin cancer detection are included in the survey article (Okur and Turkan, 2018), considering different challenges for skin cancer problems, and some research directions are provided along with. To aid comprehension, a general outline of a computer-assisted diagnosis system was revisited in Vocaturo et al. (2019). Consequently, a roadmap of classification algorithms was presented, incorporating emerging paradigms of artificial intelligence. Specifically, Multiple Instance Learning approaches and the Deep Learning paradigm were highlighted as noteworthy for implementing more robust and effective solutions. Moreover, AI algorithms are powerful but often opaque, lacking transparency and explanations for their decisions. The need for Explainable AI (XAI) is recognized, particularly in the medical field where decisions impact lives. Caroprese et al. (2022) examined the benefits of using logic approaches for XAI, focusing on argumentation theory in Medical Informatics. Three categories were identified: Argumentation for Medical Decision Making, Explanations, and Dialogues.

It is evident that, based on the extraction of quantitative features, CNNs surpass human observations. However, the main challenge with CNNs lies in their inability to differentiate between significant and insignificant biological features, including artifacts. During CNN training, correlations with the training dataset that are unnecessary may be chosen, hindering generalization (Zech et al., 2018; Lapuschkin et al., 2019; Schmitt et al., 2021). Conversely, input images are crafted with deception, capable of deceiving CNNs, a phenomenon known as adversarial attacks (Heaven, 2019). In dermatology, both scenarios are encountered (Fawzi and Frossard, 2015; Finlayson et al., 2019; Winkler et al., 2019; Zhang, 2019). In this paper, we address these issues by focusing primarily on utilizing features of dermoscopic structures, rather than typical features such as color, size, and shape. Subsequently, our models are tested on various versions of test sets to analyze their performances and assess their resilience against brittleness.

In the field of dermatology, dermatologist experts use a criteria known as the three-point checklist of dermatology, or 3PCLD, for assessing lesions, which is considered a superior method for examining lesions (Argenziano et al., 2003; Soyer et al., 2004). According to this method, the criteria for lesion assessment include asymmetry in shape, hue, and distribution of a specific structure called the blue-white veil; the presence or absence of which is indicated by specific values such as 0, 1, or 2 (Argenziano et al., 2003; Soyer et al., 2004; Carrera et al., 2016). Another criterion, the seven-point checklist of dermatology or 7PCLD, includes, in addition to the blue-white veil, various other structures like dots, globules, streaks, and regression structures, etc. (Argenziano et al., 1998; Carrera et al., 2016; Kawahara et al., 2018). To date, limited research has been conducted to explore the potential use of dermoscopic structures (Soyer et al., 2004).

## 3 Methodology

**Figure 2:**
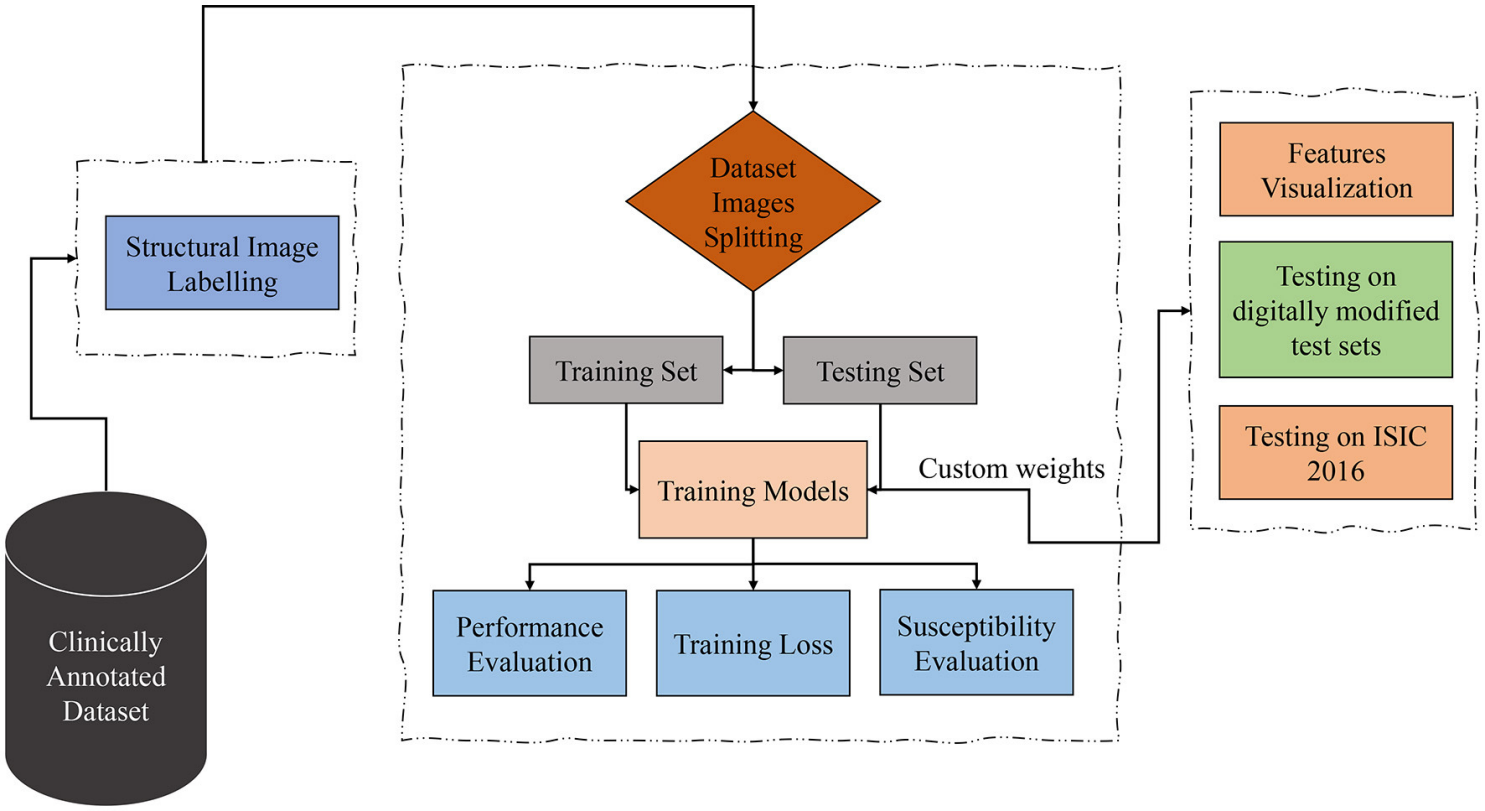
Proposed technique block diagram based on dermoscopic structure features: the clinically annotated dataset is labeled according to dermoscopic structure features, divided into training and testing sets for training models for detection based on these features. Subsequently, performance, training losses, and the susceptibility of training models to image variations are evaluated. Feature visualization is applied to determine where the model focuses during detection. The models are then tested for brittleness on different versions of test sets. In the final step, trial-and-error testing is conducted on the ISIC 2016 dataset to detect dermoscopic structures within it.

Considering the datasets containing clinically marked images by expert clinicians and dermatologists for the presence of a particular dermoscopic structure, the training images undergo processing through Transformers and CNN-based models to classify them based on the detection of dermoscopic structures as features. The results of the classification undergo validation using feature visualization or explainable AI, illustrating where the model is directing its attention. Furthermore, to assess the models' susceptibility to image variations, all classifiers under examination are evaluated on test sets comprising both original and duplicated images with digital modifications. As the transformations applied to the test sets are artificial, the models under study are also tested on ISIC 2016 images. The proposed technique is presented in Figure 2 and is discussed in detail in the subsequent subsections.

**Figure 3:**
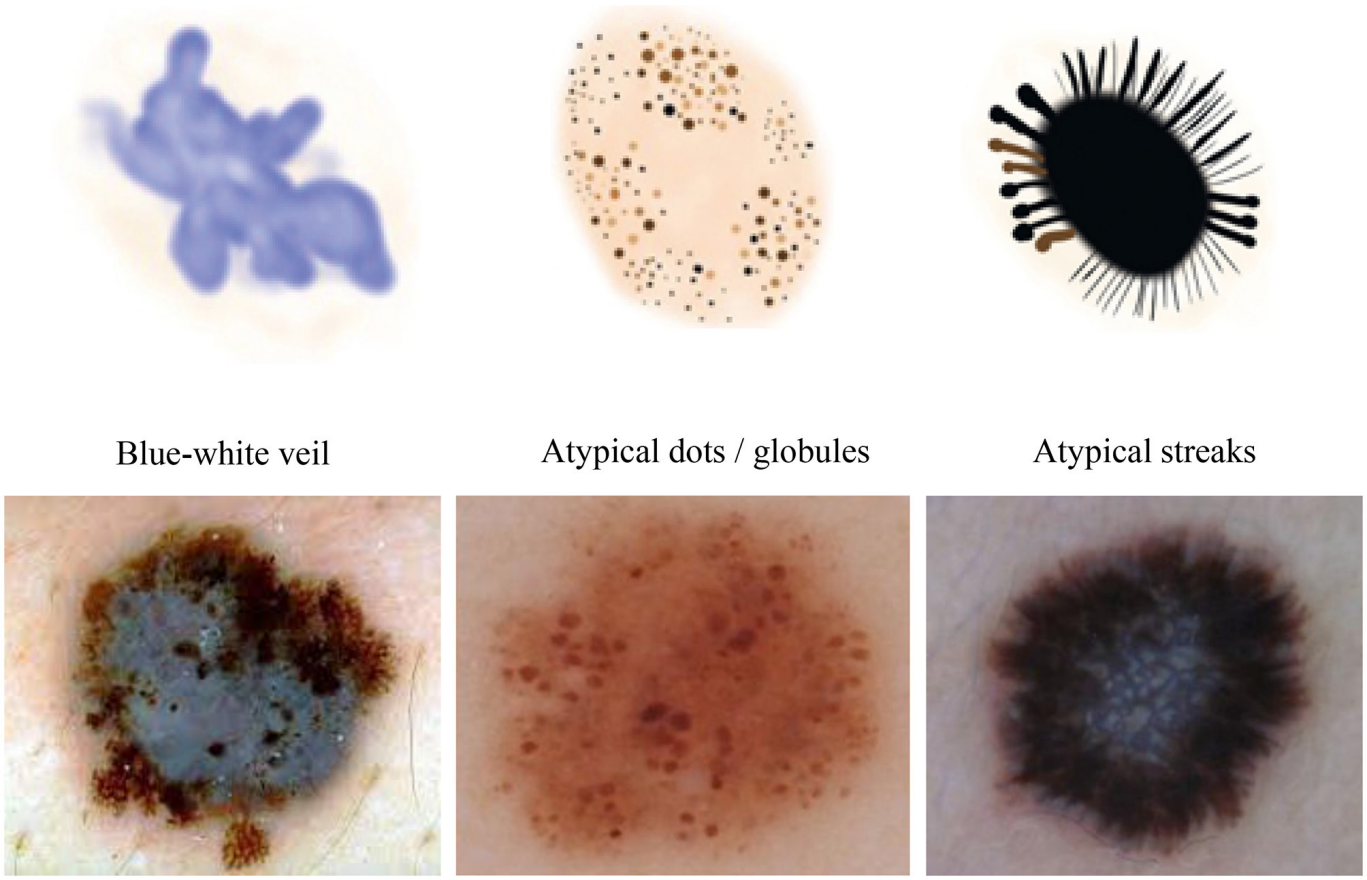
Dermoscopic structures can be observed in dermoscopic images of various melanocytic lesions, accompanied by their computerized illustrations. In our study, these dermoscopic structures are assessed based on features categorized as present, absent, regular, and irregular.

We conducted our study on three distinct dermoscopic structures, namely Blue-white veil, dots/globules, and streaks (crucial dermoscopic structures for melanoma detection and classification are depicted in Figure 1). The blue-white veil manifests as blue pigmentation in certain ill-defined areas of the lesion, while streaks represent pigmented projections at the lesion's outer margins. Globules are round to oval structures larger than 0.1mm. Figure 3 displays dermoscopic images containing these three structures, accompanied by their illustrations. In our technique, we assess these dermoscopic structures in terms of their presence, absence, and sub-categories of regular and irregular.

### 3.1 Convolutional neural networks

In this paper, to facilitate a comparison between transformers-based and CNN-based architectures, we evaluate the classification performance of several CNN architectures by utilizing dermoscopic structures as features instead of relying on typical features such as color, size, or shape. For this purpose, three widely used architectures, namely ResNet-50, VGG-16, and DenseNet-121, are considered. The CNN architectures and training techniques chosen for this study are commonly employed for melanoma classification tasks using features other than dermoscopic structures (Esteva et al., 2017; Barata et al., 2018; Yap et al., 2018; Kassani and Kassani, 2019).

#### 3.1.1 ResNet-50

ResNet-50 (He et al., 2016), a significant advancement in deep learning, has transformed the realm of convolutional neural networks (CNNs) through its revolutionary design and training approach. Created by Microsoft Research, ResNet-50 belongs to the ResNet series, bringing forth the novel idea of residual learning to address issues related to training extremely deep neural networks. ResNet-50 is distinguished by its 50 layers, surpassing its predecessors in depth. The core components of ResNet-50 are its residual blocks, which enable the network to grasp residual functions.

#### 3.1.2 VGG-16

VGG-16 (Simonyan and Zisserman, 2014), an abbreviation for Visual Geometry Group 16, stands as a convolutional neural network architecture designed specifically for image classification. Conceived by the Visual Geometry Group at the University of Oxford, VGG-16 has garnered acclaim for its simplicity and effectiveness, solidifying its status as a benchmark architecture in the realm of computer vision. The architecture of VGG-16 exhibits a consistent and structured framework, comprising a total of 16 layers, as implied by its name. This includes 13 convolutional layers, succeeded by three fully connected layers, and culminating in an output layer.

#### 3.1.3 DenseNet-121

DenseNet-121 (Huang et al., 2017), a member of the DenseNet family or Dense Convolutional Networks, presents a convolutional neural network (CNN) architecture distinct from conventional CNNs. It introduces dense connectivity patterns, where each layer receives input from all preceding layers, fostering feature reuse, parameter reduction, and improved information flow within the network. The fundamental components of DenseNet-121 are dense blocks, comprising multiple interconnected layers. Each layer receives input from all preceding layers by concatenating their feature maps. This approach promotes feature reuse, addressing the vanishing gradient problem and encouraging the network to learn more discriminative features.

### 3.2 OpenAI CLIP

Contrastive Language Image Pre-training (CLIP) (Radford et al., 2021) serves as a zero-shot classifier, utilizing English language knowledge to categorize images without necessitating prior training on a specific dataset. Exhibiting an accuracy of 41% across 400 million images, in contrast to the 16% accuracy of the Transformers model and 27% achieved by Bag of Words, CLIP demonstrates the capability of faster training in comparison to other models within the same domain. The CLIP model undergoes training across a diverse array of image styles, enabling superior generalization and flexibility compared to ImageNet. Functioning as a zero-shot detector, CLIP exhibits the ability to generalize to entities it has not encountered previously. The program is trained on an extensive database comprising both text and images, facilitating diverse tasks such as image search, caption generation, format transfer, and text-to-image generation. Although still in its early stages, the CLIP project harbors the potential to become a pivotal tool across a broad spectrum of applications. Employing a contrastive learning objective, CLIP learns to discern between connected and unconnected pairs of images and text. For specific tasks, CLIP can undergo refinement aimed at enhancing its performance, thus presenting a robust tool applicable to various undertakings. Despite being under development, CLIP emerges as a promising resource for both researchers and developers.

**Figure 4:**
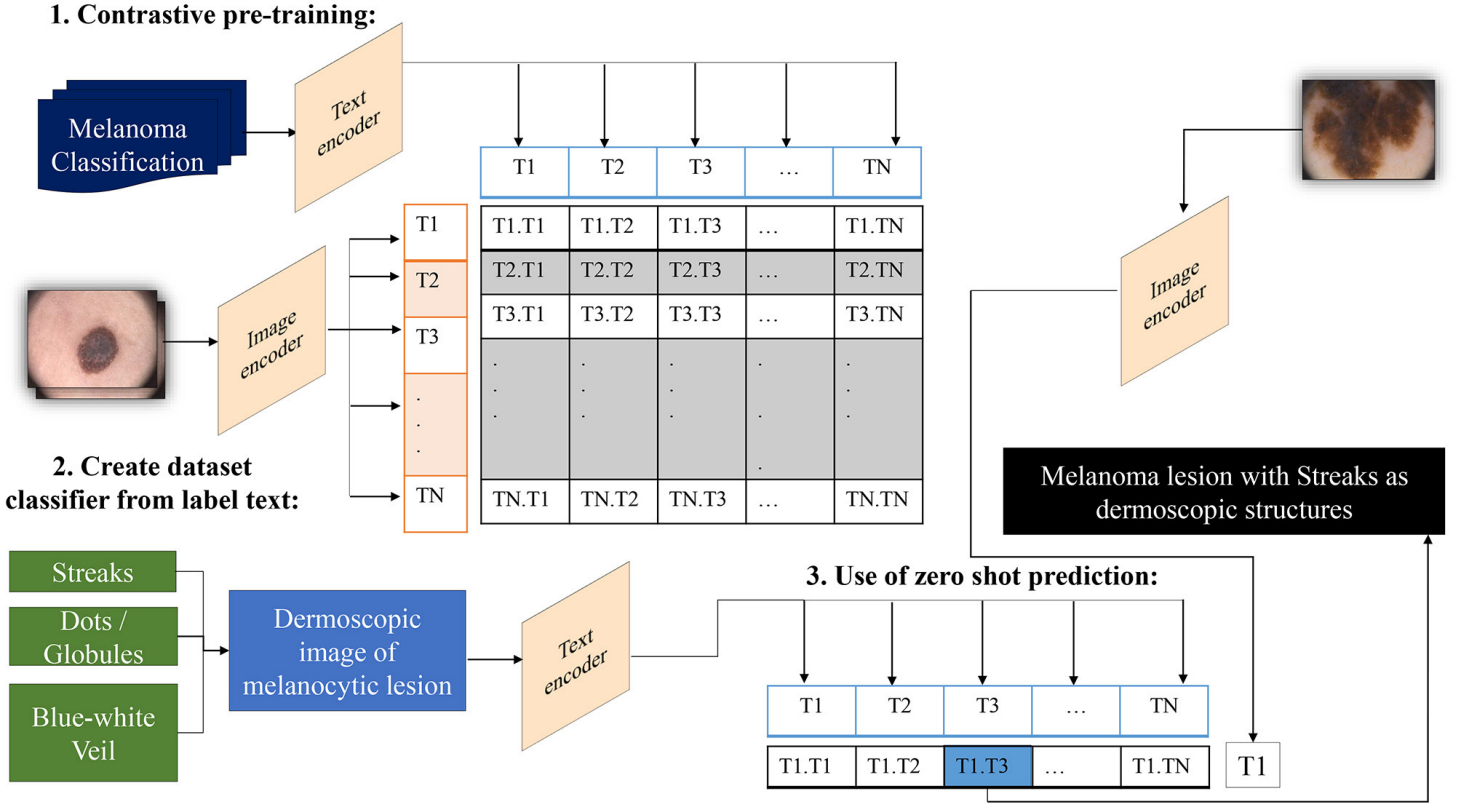
Overview of the zero-shot detector as a melanoma classifier based on dermoscopic structures as features: Textual prompts are processed by the text encoder, a language model, while input images are handled by the image encoder, a CNN model. Both text and image encoders produce vector representations for text and images, respectively. Subsequently, the similarity between the vector representations of text and images is computed through contrastive loss during pre-training. This process endows CLIP with the capability to generate a descriptive caption for an image. Conversely, CLIP can retrieve images that match the textual prompt provided to it.

In traditional classifiers, integers replace class labels internally. CLIP generates an encoding for its text-to-image pairs. Therefore, to classify the presence of dermoscopic structures, the ability to encode classes utilizes transformers' capacity to classify images by extracting meaning from text without the need to fine-tune custom data. All that is required is to define a list of potential classes, along with necessary image descriptions, etc., and CLIP will classify images belonging to specific classes based on its prior knowledge. In other words, we can interpret this as asking the model which captions best match the given images (Radford et al., 2021). The operation of a zero-shot detector in terms of the structural classifier is illustrated in Figure 4.

CLIP establishes a collective embedding space where the representations of images and corresponding text are close when they express comparable semantic meanings. This space is deliberately trained to carry semantic significance, empowering the model to understand the connection between images and text. In the context of zero-shot melanoma detection using CLIP, the procedure involves defining the task by supplying a textual prompt that characterizes the dermoscopic structure targeted for detection. Subsequently, CLIP generates visual embeddings based on this prompt and examines its embedding space to identify images demonstrating semantic relevance to the specified concept. The model generates a similarity score for each image in the dataset in relation to the provided textual prompt. Applying a threshold to this score allows the identification of images where the model suggests the presence of the specified structure. Adjusting this threshold provides control over the balance between precision and recall.

### 3.3 Vision Transformers

Vision Transformers (ViTs) are a type of artificial neural network with the capability to be utilized in computer vision tasks, such as image classification and object detection. ViTs are built upon the transformer architecture, originally designed for tasks related to natural language processing. Transformers analyze the various components of an input sequence and learn to predict the next words in those sequences. ViTs follow a similar approach, but instead of focusing on words, they examine different parts of an image. Demonstrations have shown that ViTs are highly effective for a variety of computer vision tasks, particularly excelling in image classification tasks such as the ImageNet dataset, achieving a remarkable accuracy of 84.4%. ViTs do not necessitate spatial convolutions, which can be computationally expensive. Moreover, scaling ViTs to larger models does not compromise accuracy. ViTs prove beneficial for specific tasks like image classification, object detection, or segmentation. Motivated by their recent performance advancements, we have selected ViTs for our study.

**Figure 5:**
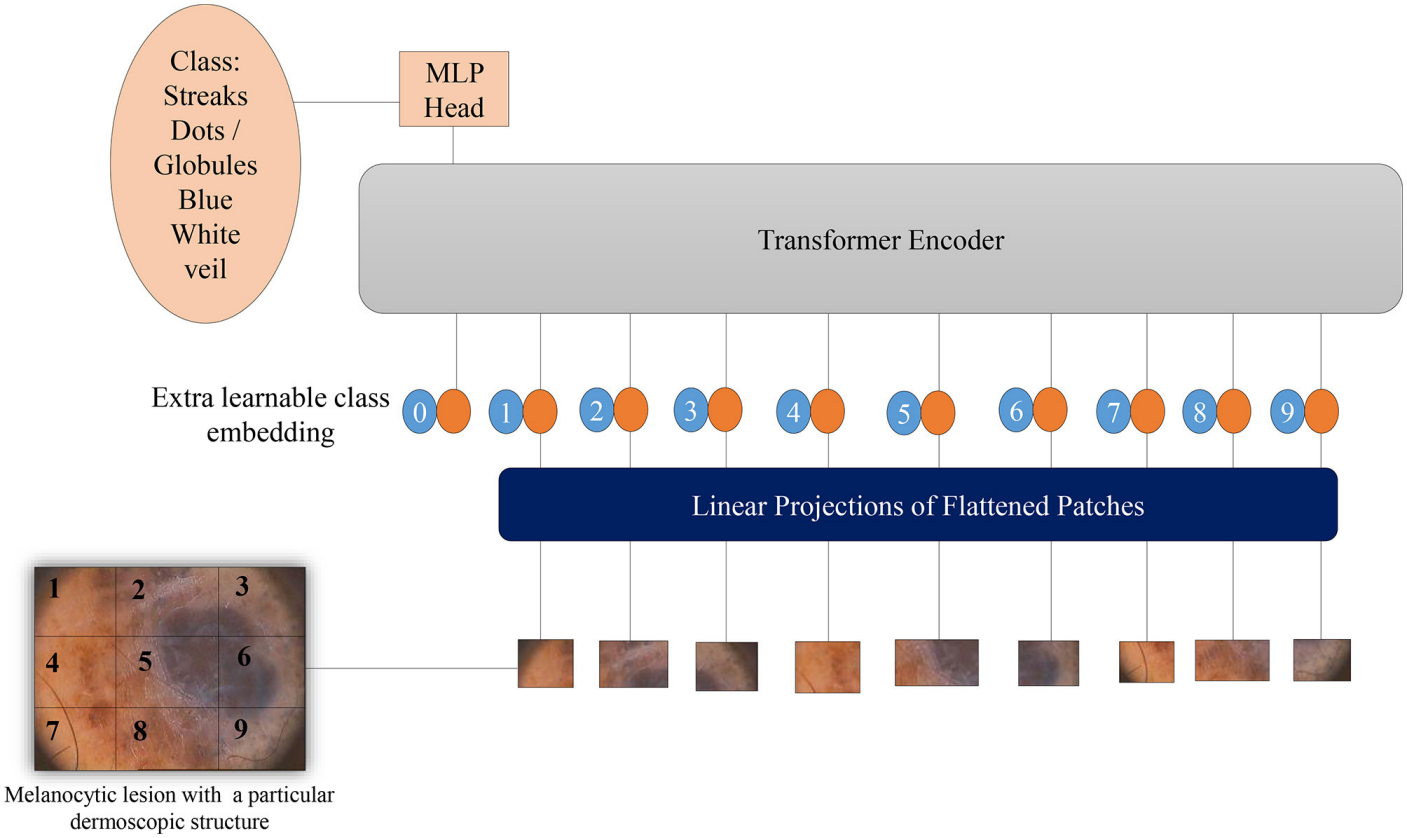
Overview of the architecture of Vision Transformers for melanoma classification based on dermoscopic structures as features: The input image is divided into a grid of patches. These patches are then embedded in vector form, which is subsequently fed into the transformer encoder—a stack of self-attention layers. The transformer encoder attends to all patches in the input image, regardless of their positions. The final output class label is generated from the classification head, represented by a linear layer.

Vision Transformers (Dosovitskiy et al., 2020) leverage potent natural language embeddings for images. When input images are introduced to the model, they undergo splitting into linearly embedded patches, followed by the addition of positional embeddings. Subsequently, these patches are sequentially inputted into the transformer encoder. To facilitate image classification based on the presence of dermoscopic structures, a class token is appended at the beginning of each image sequence. Figure 5 illustrates the architecture of the Vision Transformer model for melanoma classification, considering dermoscopic structures as features. The hyperparameters employed for ViTs in our experimentation have been elucidated in the results section.

### 3.4 Introducing convolutions to Vision Transformers

Two fundamental modifications have been implemented in Vision Transformers (Wu et al., 2021): the incorporation of convolutions in a new token embedding block, and the utilization of a transformer-convolution block that capitalizes on projections through convolutions. These alterations imbue Transformers with the distinctive traits of Convolutional Neural Networks.

**Figure 6:**
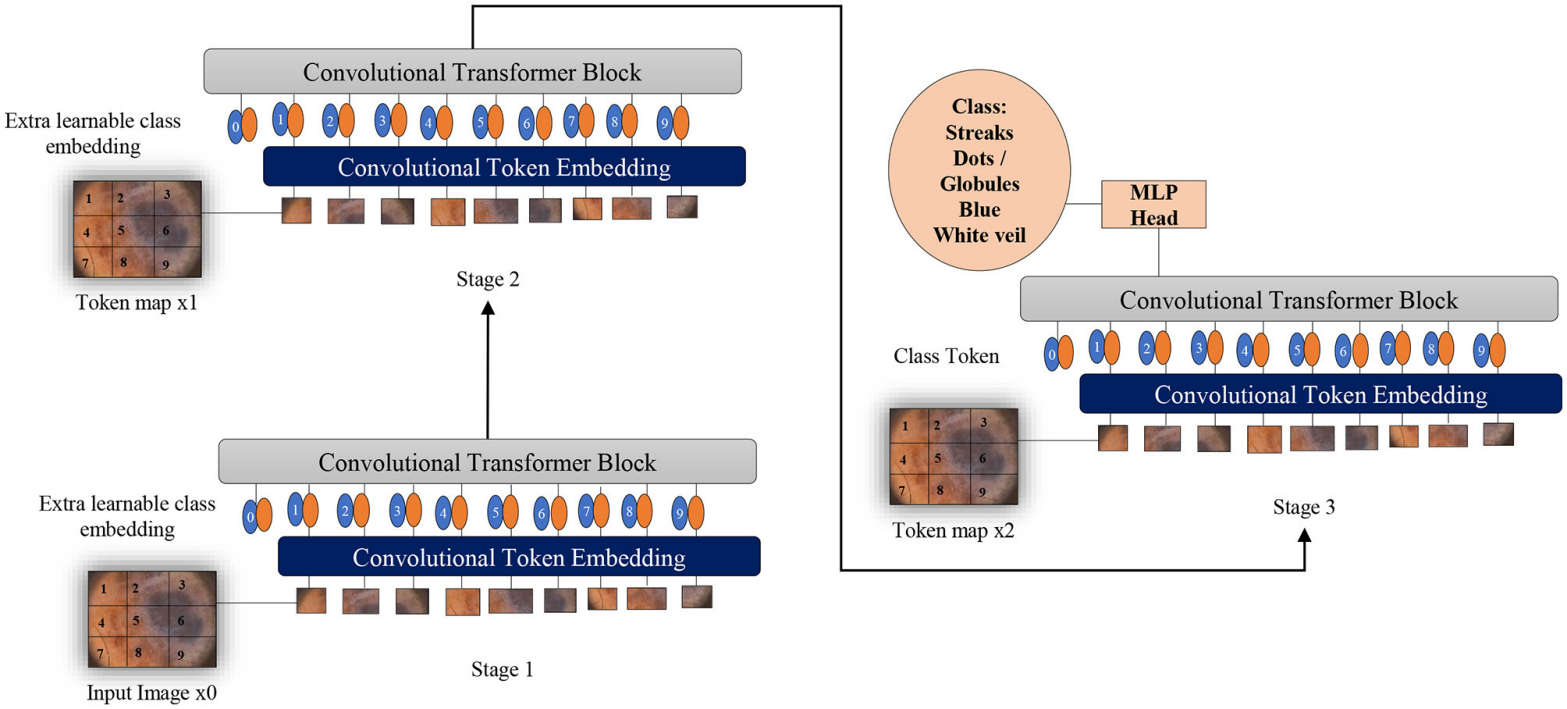
Overview of applying convolutions to Vision Transformers as melanoma classifiers based on dermoscopic structures as features: an additional convolution layer is employed to transform patches of input images into vectors. A supplementary convolutional transformer block is utilized to ascertain the similarity among patches that are in close proximity within the input image. This entire process is segmented into multiple stages to produce token maps.

As depicted in Figure 6, the design borrowed from CNNs incorporates a multi-stage hierarchy consisting of a total of 3 stages, each comprising 2 parts. In the initial stage, the input image undergoes processing in the convolutional embedding layer, where convolution operations are applied using overlapping patches. The tokens are then reshaped into 2D grids mirroring the input image, followed by the application of layer normalization. Layer normalization serves to decrease the number of tokens, referred to as feature resolution, and augment their width, known as feature dimension. This aids in downsampling the spatial representation, akin to the CNNs design. The remainder of each stage encompasses a Convolution-Transformer Block, wherein a distinct depth-wise convolutional operation is employed instead of a linear projection as seen in Vision Transformers. The ultimate token for classification is introduced solely at the conclusion of the last stage, with a fully connected MLP head attached to the classification token. This MLP head connects to the output of the final stage for the purpose of predicting classes.

Given the 2D input image or 2D token map from the previous stage, convolution is applied to obtain the new token map, with the height and width being determined by:


$$H_{i}=\left\lfloor \frac{H_{i}-1+2_{p}-s}{s-o}+1\right\rfloor ,W_{i}=\left\lfloor \frac{W_{i}-1+2_{p}-s}{s-o}+1\right\rfloor$$

Where, *s* is the size of the kernel, *s*−*o* is stride and *p* is padding.

### 3.5 Methods to reduce brittleness of models

**Figure 7:**
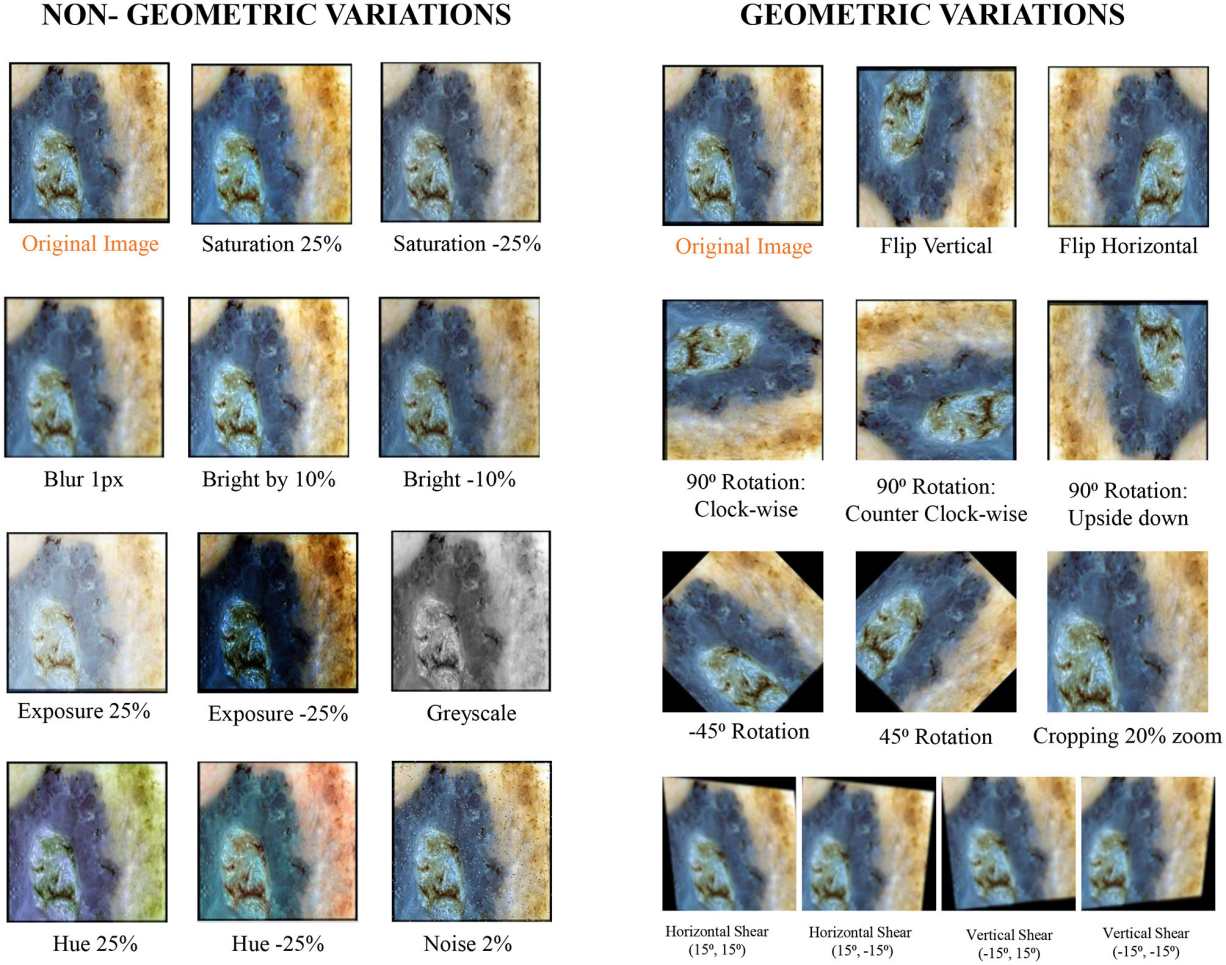
Exemplary section of images with their transformed counterparts: the specified transformations are applied to each image in the dataset, which is subsequently grouped into geometric and non-geometric variation categories.

To enhance the effectiveness of the models under study against brittleness caused by the utilization of dermoscopic structures as features, two methods were tested: dataset augmentation and test time augmentation. During the training phase, an extensive level of data augmentation was applied to the training sets. The types of transformations applied were those commonly encountered in daily clinical examinations of skin lesions. In test time augmentation, 23 transformed duplicates, in addition to the input image (a total of 24 images), were evaluated during the inference stage, and their average was considered as the final result. The transformed duplicates for a single test image are illustrated in Figure 7 and are further discussed in detail in the results section.

In deep learning, the original dataset is sometimes expanded by generating multiple copies of the original data. The tools employed for data augmentation create unique, fresh duplicates of the data by altering certain parameters of the original dataset. While data augmentation can be applied to various inputs such as text, audio, and video, our study specifically focuses on augmenting image-based datasets. There are two types of data augmentation: offline and online. In offline data augmentation, augmented images are combined with the original data and stored on a disk drive. In the online mode of data augmentation, augmentation steps are randomly applied to selected images, which are then combined with the original data for training purposes. In our study, we utilized data augmentation in offline mode, applying augmentation steps to the entire original dataset, resulting in the dataset being expanded up to 23 times, with each image having 23 copies.

The applied transformations were divided into two categories: geometric and non-geometric variations. They include (as shown in Figure 7):

- Geometric variations: Crop: 0% Minimum Zoom, 20% Maximum Zoom, Flip: Horizontal, Vertical, Rotation: Between –45° and +45°, 90 Rotate: Clock-wise, Counter-Clockwise, Upside Down, and Shear: 15° Horizontal, 15° Vertical.
- Non-Geometric variations: Blur: Up to 5px, Brightness: Between –10% and +10%, Exposure: Between –25% and +25%, Grayscale: Applied to 100% of images, Hue: Between –25° and +25°, Noise: Up to 5% of pixels and Saturation: Between –25% and +25%.

The augmented dataset was then utilized for training to mitigate the brittleness observed in the models, as discussed in the results section. Data augmentation, for example, represents one of several methods through which we can expand the size of the original dataset. Additionally, it can serve as a tool for regularization, enhancing the models' resilience to minor variations in input images.

There are various ways to enhance the performance of deep learning models by modifying the training procedures. As previously discussed, one such method is data augmentation. However, improvements can also be achieved by altering how we evaluate models. Test time augmentations represent one such approach. Similar to the impact of data augmentation on training sets, test time augmentation involves modifying test images presented to the model during evaluation. Instead of presenting only the original test image to the model for result calculation, multiple copies of the input image are shown to the model, each digitally altered. Consequently, artificial modifications employed for test time augmentation precisely mirror those applied to the original dataset for data augmentation. These modifications are detailed in the preceding subsection and depicted in Figure 7. Predictions for all digitally modified versions of a particular image are then averaged to derive a final prediction.

## 4 Experiments and results

### 4.1 Datasets details

We explored the Ph2 (Mendonça et al., 2013) and Derm7pt (Kawahara et al., 2018) datasets, which consist of RGB dermoscopic images marked with the presence or absence of certain dermoscopic structures by expert clinicians. To train and evaluate the models under study, these RGB dermoscopic images, along with their labels, were used as training data. We classified the training datasets according to clinical markings for dermoscopic structures and passed them to Vision Transformers (Dosovitskiy et al., 2020), OpenAI CLIP (Radford et al., 2021), and CNN-based architectures for model learning. The details of datasets, organized according to the features of dermoscopic structures, are presented in Table 1.

**Table 1:** Clinically annotated dermoscopic structures based datasets used in our experiments.

**Datasets**	**Training images**	**Blue-white veil**	**Dots/globules**	**Streaks**
Ph2 (Mendonça et al., 2013)	200 Dermoscopic	164 (A), 36 (P)	87 (A), 59 (AT), 54 (T)	170 (A), 30 (P)
Derm7pt (Kawahara et al., 2018)	1,006 Dermoscopic	812 (A), 194 (P)	228 (A), 447 (Irr), 332 (R)	649 (A), 250 (Irr), 106 (R)

A, Absent; P, Present; Irr, Irregular; R, Regular; AT, Atypical; T, Typical.

### 4.2 Experimental setup

All classifiers under study (Vision Transformers, OpenAI CLIP, ResNet-50, VGG-16, and DenseNet-121) were trained and evaluated using the same training and testing sets, as well as protocols. All experimentation was conducted in Python 3.7.7, utilizing Fastai in conjunction with PyTorch on online-available GPU machines.

The Vision Transformer model (Dosovitskiy et al., 2020) can be divided into three layers: ViTModel, provided by the transformers library, serves as the base model. Additionally, there is a dropout layer used for regularization, and a linear layer that takes the input image size, equal to the number of hidden nodes on the ViTModel. The output of this linear layer corresponds to the number of classes. The linear layer functions as the final layer (Dosovitskiy et al., 2020). The ViTModel employed for Vision Transformers is a standard PyTorch model, utilizing a dropout value of 0.1. We conducted training for our Vision Transformers over 30 epochs, employing a batch size of 10. The learning rate was set to 2*e*^−5^, and we utilized a custom ViT feature extractor in conjunction with the Adam optimizer. As a pre-processing step, all images were rescaled to dimensions of 224 × 224. Convolutions were introduced to Vision Transformers through CvT-13, a basic model consisting of 19.98 million parameters.

CLIP functions as a zero-shot image classifier, indicating that it requires no training. For classification oncology, experiments have been conducted using various class captions. CLIP was pre-trained to distinguish between images and caption pairs. In the realm of vision, CLIP incorporates recent advancements, such as those seen in large-scale transformers like GPT-3.

ResNet-50, VGG-16, and DenseNet-121 are convolutional neural networks commonly employed for image classification tasks. As suggested by their names, ResNet-50 comprises 50 layers, VGG-16 consists of 16 layers, and DenseNet-121 incorporates 121 layers. For all 3 models kernel size of 3 × 3 is used and we have used cross-entropy as the loss function. These models have been implemented using Fastai, a low-code deep learning framework.

In the initial phase, all models under study were trained on natural datasets without applying any augmentations and were tested on various versions of test sets. The training datasets were divided in an 80:20 ratio into training and validation sets. The artificial test sets were duplicated 23 times, and the applied transformations included cropping, flipping, rotation, and changes in brightness, among others. All these transformations were divided into two categories: geometric and non-geometric variations. The cropped test sets were analyzed manually afterward to ensure that the lesion information wasn't compromised. Moreover, testing was also conducted on the natural test sets and on combined test sets that included all transformations. Some exemplary images from the natural and artificial test sets, along with the applied transformations, are shown in Figure 7. After the calculation of results, methods were applied to reduce the brittleness exhibited by a few models under study, as discussed in the methodology section.

### 4.3 Evaluation metrics: loss and accuracy

Choosing loss function is significantly important in dermoscopic structures-based melanoma lesion classification as there is an imbalance between the samples of positive and negative classes. By not considering the class imbalance in the loss function, the model can diverge to a sub-optimal solution. Moreover, in terms of medically applicated CAD-based systems, reductions in false positive predictions are always preceded by reductions in false negative predictions. Concretely, predicting the presence of dermoscopic structures is more important than falsely predicting their absence. Therefore, we have used cross-entropy loss in our experimentation.

Cross entropy or log loss, is used to analyze the performance of classification models whose output values lie within the probability range of 0 and 1. This cross-probability increases as the model prediction diverges from the actual value. Log loss and cross-entropy loss are somewhat different up to a certain extent but in machine learning or deep learning, for calculation of errors between 0 and 1, they almost conclude to the same thing. Cross entropy loss is given by:


$$L_{CE}=-\overset{n}{\underset{i=1}{\sum }}t_{i}log\left(p_{i}\right)$$

for n classes.

Where *t*_*i*_ is truth label and softmax probability for the *i*^*th*^ class is *p*_*i*_.

The performance of the classification tasks was assessed using the cross-entropy loss function and accuracy (A). Initially, these metrics were calculated for each class of dermoscopic structures separately for both datasets, and the final result was obtained by averaging them. The aforementioned criteria is defined as follows:


$$A=\frac{TP+TN}{TP+TN+FP+FN}$$

Here, TP (True Positive) represents the samples predicted and classified as actually true based on dermoscopic structures as features. FP (False Positive) denotes the samples predicted as true but classified as wrong positives, while TN (True Negative) includes samples predicted as false and are indeed false.

### 4.4 Results and discussion

In this subsection, we present the findings from our classification tasks, which are based on the models under investigation. We utilize dermoscopic structures as features for assessing Melanoma Lesions. To achieve this, we provide a comprehensive report for each model, detailing its training on natural datasets and testing on various versions of test sets. Subsequently, we present the outcomes of all models by considering the collective impact of the three dermoscopic structures. Additionally, we conduct an analysis to identify where the model focuses during classification, employing Grad-cams. In the following stage, we implement methods to mitigate brittleness observed in models across diverse versions of artificially transformed test sets. We then compare the results obtained from both stages.

#### 4.4.1 Classification results using dermoscopic structures as features

The dermoscopic structures under study have already been discussed in the literature section. The results of classification for melanocytic lesions, considering these structures, are shown in Table 2. The table represents the cross-entropy loss and accuracy of classification obtained from all models against each dataset under study. To quantify prediction accuracy, the accuracy for each individual category is calculated.

**Table 2:** Results of classification obtained on natural testsets: STATS NATURAL.

**Models (averaged for all 3 dermoscopic structures)**	**Datasets**	**Accuracy**	**Training loss**
ResNet-50	PH2	0.68	1.52
VGG-16	PH2	0.48	1.32
DenseNet-121	PH2	0.45	1.05
OpenAI CLIP	PH2	0.51	–
**Vision Transformers**	**PH2**	**0.94**	**0.53**
**Vision Transformers with convolutions**	**PH2**	**0.95**	**0.50**
ResNet-50	DERM7	0.6	0.77
VGG-16	DERM7	0.59	0.86
DenseNet-121	DERM7	0.66	0.76
OpenAI CLIP	DERM7	0.43	–
**Vision Transformers**	**DERM7**	**0.96**	**0.59**
**Vision Transformers with convolutions**	**DERM7**	**0.97**	**0.53**

The bold selections have been highlighted to showcase our implemented technique/model or to underscore its exceptional accuracy.

For the blue-white veil, results are predicted for the subcategories of presence and absence, while streaks and dots/globules are tested in terms of absence, regular, and irregular subcategories. The result accuracies of all dermoscopic structures under study are then averaged to calculate the final results.

**Figure 8:**
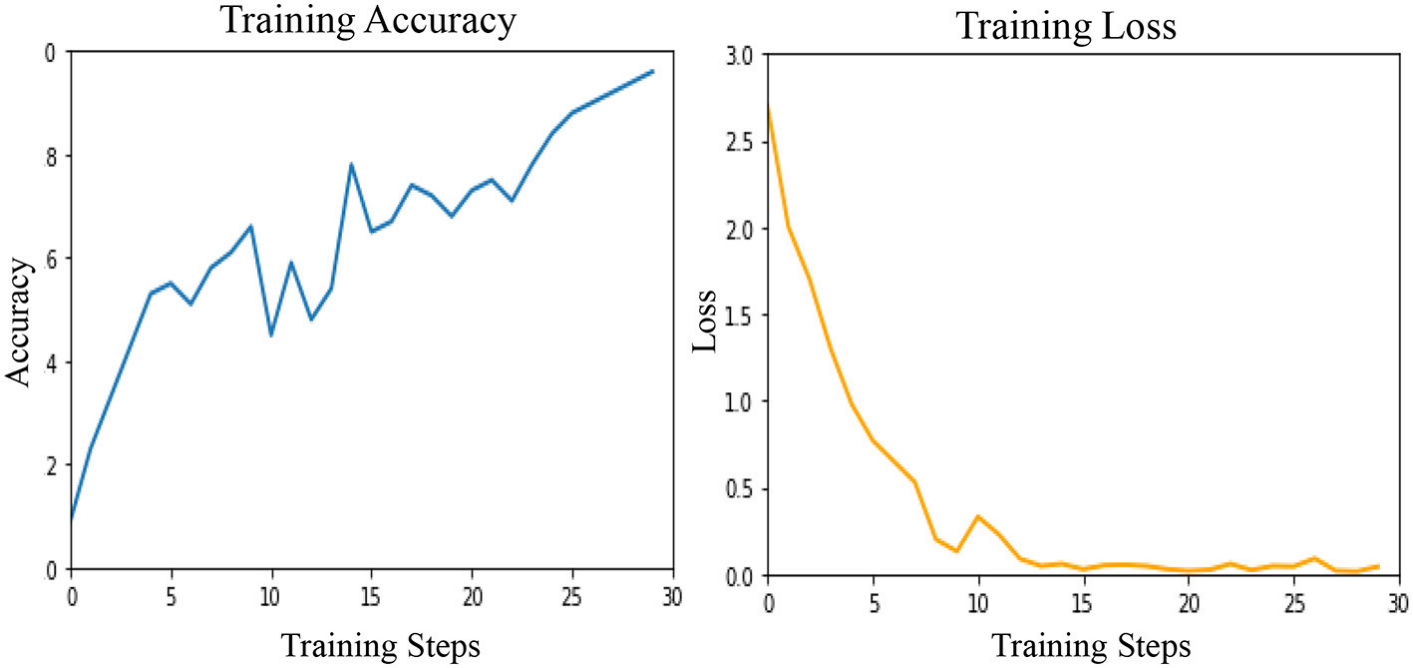
Training accuracy and loss graphs as obtained by Vision Transformers.

These results are presented in Table 2 as STATS NATURAL. The table shows that introducing convolutions to Vision Transformers provides the highest accuracy when dermoscopic structures are used as features for both datasets. However, the accuracies depicted by CNN architectures, particularly VGG-16 and DenseNet-121, range between 50–60%, indicating the highest accuracies when features other than dermoscopic structures are used. In Table 2, the results are stated for models according to the datasets under study. However, the training loss and accuracy graphs obtained by training Vision Transformers are shown in Figure 8.

#### 4.4.2 Testing on varied versions of artificially transformed testsets

To assess the susceptibility of all models under study, the models trained on natural datasets are subjected to testing using artificially varied versions of test sets. These artificial transformations simulate conditions commonly encountered in the clinical examination of lesions. Tables 3, 4 present the classification results obtained through testing on diverse versions of test sets. These varied versions are categorized into two groups: Geometric variations (Crop: 0% Minimum Zoom, 20% Maximum Zoom, Flip: Horizontal, Vertical, Rotation: Between –45° and +45°, 90°° Rotate: Clock-wise, Counter-Clockwise, Upside Down and Shear: 15° Horizontal, 15° Vertical) and Non-Geometric variations (Blur: Up to 5px, Brightness: Between –10% and +10%, Exposure: Between –25% and +25%, Grayscale: Apply to 100% of images, Hue: Between –25° and +25°, Noise: Up to 5% of pixels and Saturation: Between –25% and +25%). The testing results for classification based on the features of dermoscopic structures in these geometrically and non-geometrically varied test sets are presented in Tables 3, 4, respectively.

**Table 3:** Results of classification obtained on geometrically varied testsets: STATS GEOMETRIC VARIATIONS.

**Models (averaged for all 3 dermoscopic structures)**	**Datasets**	**Accuracy**
ResNet-50	PH2	0.64 ↓
VGG-16	PH2	0.54 ↑
DenseNet-121	PH2	0.63 ↑
OpenAI CLIP	PH2	0.40 ↓
Vision Transformers	PH2	0.89 ↓
Vision Transformers with convolutions	PH2	0.96 ↑
ResNet-50	DERM7	0.62 ≈
VGG-16	DERM7	0.63 ↑
DenseNet-121	DERM7	0.65 ≈
OpenAI CLIP	DERM7	0.41 ≈
Vision Transformers	DERM7	0.89 ↓
Vision Transformers with convolutions	DERM7	0.97 ≈

The text in red signifies a decrease in accuracy. The text in green indicates either an increase or maintained accuracy.

**Table 4:** Results of classification obtained on geometrically varied testsets: STATS NON-GEOMETRIC VARIATIONS.

**Models (averaged for all 3 dermoscopic structures)**	**Datasets**	**Accuracy**
ResNet-50	PH2	0.58 ↓
VGG-16	PH2	0.55 ↑
DenseNet-121	PH2	0.59 ↑
OpenAI CLIP	PH2	0.40 ↓
Vision Transformers	PH2	0.87 ↓
Vision Transformers with convolutions	PH2	0.96 ↑
ResNet-50	DERM7	0.61 ↑
VGG-16	DERM7	0.66 ↑
DenseNet-121	DERM7	0.64 ≈
OpenAI CLIP	DERM7	0.41 ≈
Vision Transformers	DERM7	0.90 ↓
Vision Transformers with convolutions	DERM7	0.97 ≈

The text in red signifies a decrease in accuracy. The text in green indicates either an increase or maintained accuracy.

It is evident from Tables 2–4 that classification accuracy deteriorates on varied versions of test sets when tested using Vision Transformers, and remains approximately the same or increases for some CNN-based architectures, including VGG-16 and DenseNet-121. When convolutions are applied to Vision Transformers, the accuracy increases for the Ph2 dataset and is equal for a dataset of Derm7pt. OpenAI Clip provides approximately the same accuracies on all-natural, geometric, and non-geometric varied test sets, as the Clip is a pre-trained model and predicts the results based on matching image-to-caption pairs. These three tables show that Vision Transformers (without convolutions) are more prone to AI variations, predicting the brittleness of the model.

#### 4.4.3 Localization/explainable AI

**Figure 9:**
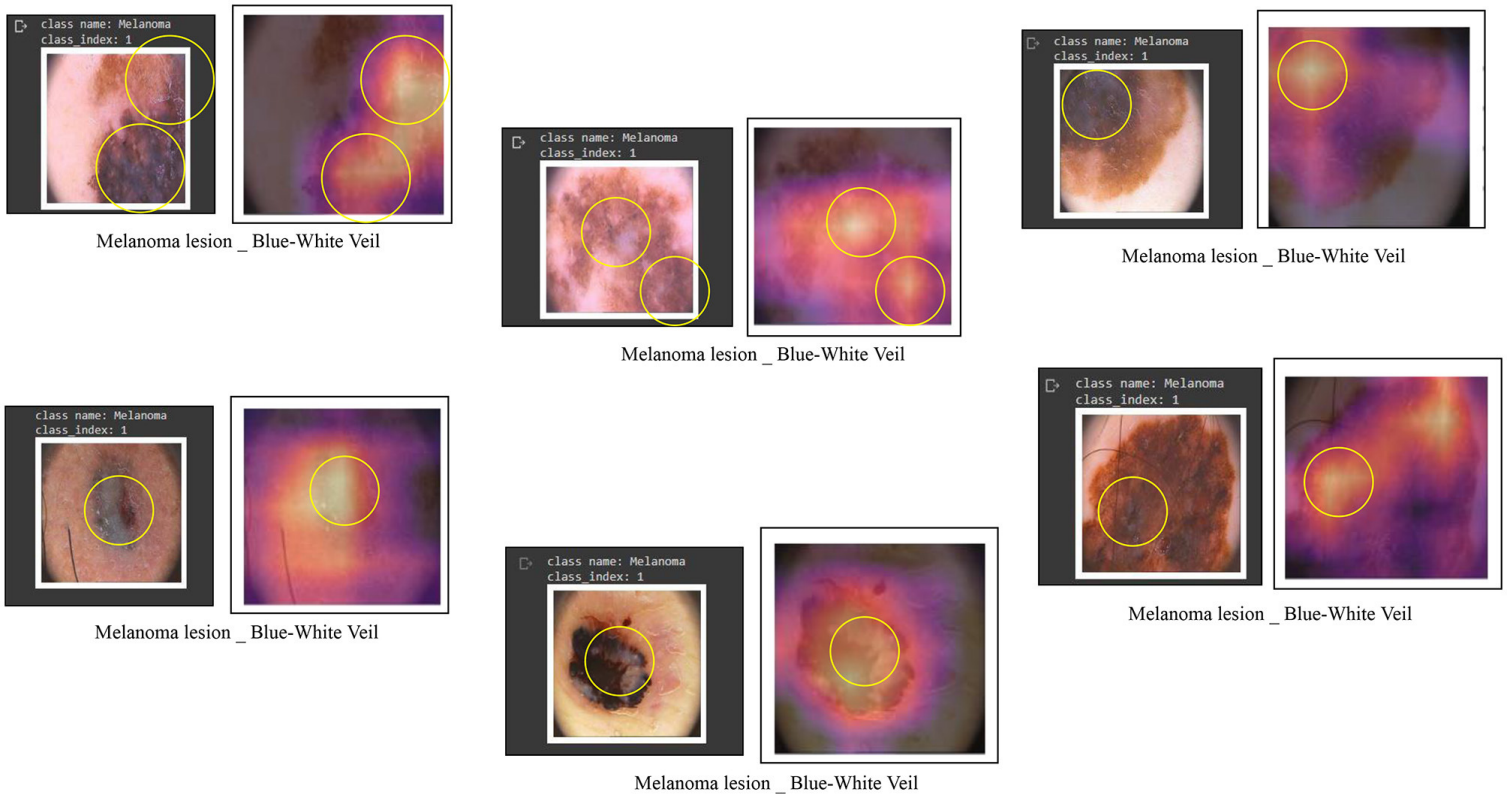
Some examples of feature visualization for blue white veil showing corresponding regions considered for classification by Vision Transformers.

To develop a classification model with outputs that are understandable to humans, the visualization of image areas is crucial in contributing to label predictions. This involves examining the learned *h* × *w* responses that impact the outcome of a specific *l*^*th*^ label. For instance, Figure 9 illustrates classification responses related to the blue-white veil. To ascertain the presence of these features, users can visualize these influential areas, thereby enhancing their confidence in deep learning models. For this visualization purpose, we employed a class activation maps-based approach, specifically known as Grad-CAMs (Zhou et al., 2016). Notably, this technique offers the additional advantage of inferring labels from images labeled by experts, surpassing reliance solely on a classification system and proving more interpretable than other localization or classification systems.

#### 4.4.4 Results of methods applied to reduce brittleness of models

As demonstrated in Tables 2–4, Vision Transformers (without convolutions) exhibit greater susceptibility to brittleness, characterized by a decline in performance when subjected to artificial changes in the test images. Consequently, to enhance the resilience of the models under investigation against brittleness induced by dermoscopic structures used as features, two methods were assessed: dataset augmentation and test time augmentation, extensively discussed in the methodology section. During the training phase, an extensive level of data augmentation is applied to the training sets, employing transformations commonly encountered in daily clinical examinations of skin lesions. In test time augmentation, 23 transformed duplicates, along with the input image, are evaluated at the inference stage, and their average is considered the final result, as illustrated in Figure 7.

The outcomes obtained by applying these two augmentation methods, namely data augmentation and test time augmentation, in the context of Vision Transformers and OpenAI Clip for various versions of natural test sets are presented in Tables 5–7. The Tables 5–7 clearly indicate that the accuracy of the results does not deteriorate when tested on multiple versions of test sets, thanks to the application of these two methods to mitigate brittleness. Notably, OpenAI Clip exhibits increased accuracy, benefiting from its lack of prior training requirements, while data augmentation leads to an expanded dataset, ultimately contributing to heightened accuracy. In the case of Vision Transformers, the training loss remains consistent when augmentations are applied, mirroring the scenario observed in training without augmentations.

**Table 5:** Results of classification obtained on natural testsets by applying dataset augmentation and test-time augmentation: STATS NATURAL.

**Models (averaged for all 3 dermoscopic structures)**	**Datasets**	**Accuracy**
OpenAI CLIP	PH2	0.55
**Vision Transformers**	PH2	**0.96**
OpenAI CLIP	DERM7	0.46
**Vision Transformers**	DERM7	**0.98**

The bold selections have been highlighted to showcase our implemented technique/model or to underscore its exceptional accuracy.

**Table 6:** Results of classification obtained on geometrically varied testsets by applying dataset augmentation and test-time augmentation: STATS GEOMETRIC VARIATIONS.

**Models (averaged for all 3 dermoscopic structures)**	**Datasets**	**Accuracy**
OpenAI CLIP	PH2	0.56 ↑
Vision Transformers	PH2	0.95 ≈
OpenAI CLIP	DERM7	0.57 ↑
Vision Transformers	DERM7	0.97 ≈

The text in green indicates either an increase or maintained accuracy.

**Table 7:** Results of classification obtained on geometrically varied testsets by applying dataset augmentation and test-time augmentation: STATS NON-GEOMETRIC VARIATIONS.

**Models (averaged for all 3 dermoscopic structures)**	**Datasets**	**Accuracy**
OpenAI CLIP	PH2	0.55 ≈
Vision Transformers	PH2	0.94 ≈
OpenAI CLIP	DERM7	0.47 ↑
Vision Transformers	DERM7	0.96 ≈

The text in green indicates either an increase or maintained accuracy.

### 4.5 Comparisons of results

In this subsection, we compare the results of all models utilized in our experimentation based on the individual dermoscopic structures under study. The accuracy obtained for each dermoscopic structure is subsequently averaged to calculate the final result for a particular model and dataset, as depicted in Tables 2–7. This detailed comparison will be discussed in the upcoming sections.

#### 4.5.1 Ablation study of all models under study on features of dermoscopic structures

We conducted an ablation study comparing CNN, CLIP, and Vision Transformers to identify the optimal dermoscopic detector for melanoma structures. Detecting melanoma poses a challenge due to the diverse natural and artificial variations present in the dermoscopic images under examination. Therefore, we maintained a consistent experimental environment and assessed the effects of various versions of each model on the study of melanoma dermoscopic structures. The Vision Transformers model yielded superior results, leveraging robust natural language embeddings for images. The introduction of convolutions to Transformers further enhanced accuracy. In contrast, CLIP functions as a zero-shot classifier, utilizing knowledge of the English language to classify images without the need for prior training on a specific dataset. The comparison results for each dermoscopic structure studied, encompassing datasets such as Ph2 and Derm7pt, are presented in Table 8.

**Table 8:** Ablation study of all models on natural versions of testsets after applying methods to reduce brittleness: STATS NATURAL.

**Models**	**Datasets**	**Structure and accuracy**
ResNet-50	PH2	BW: 0.65, D/G: 0.5, Sks: 0.88
VGG-16	PH2	BW: 0.75, D/G: 0.45, Sks:0.25
DenseNet-121	PH2	BW: 0.83, D/G: 0.28, Sks: 0.25
OpenAI CLIP	PH2	BW: 0.63, D/G: 0.27, Sks: 0.75
**Vision Transformers**	**PH2**	**BW: 1.00, D/G: 0.87, Sks: 1.00**
**Vision Transformers with convolutions**	**PH2**	**BW: 1.00, D/G: 0.89, Sks: 1.00**
ResNet-50	DERM7	BW: 0.62, D/G: 0.52, Sks: 0.67
VGG-16	DERM7	BW: 0.80, D/G: 0.48, Sks: 0.49
DenseNet-121	DERM7	BW: 0.82, D/G: 0.55, Sks: 0.61
OpenAI CLIP	DERM7	BW: 0.4, D/G: 0.37, Sks: 0.61
**Vision Transformers**	**DERM7**	**BW: 1.00, D/G: 0.97, Sks: 0.97**
**Vision Transformers with convolutions**	**DERM7**	**BW: 1.00, D/G: 0.97, Sks: 0.98**

BW, Blue-White Veil; D/G, Dots/Globules; Sks, Streaks. The bold selections have been highlighted to showcase our implemented technique/model or to underscore its exceptional accuracy.

To gain a deeper understanding of the specific transformations impacting each model's degraded performance, we conducted a thorough analysis of each applied transformation individually. This approach offers valuable insights into the unique challenges encountered by AI models in distinguishing melanoma from benign lesions and facilitates the development of targeted strategies to enhance their performance.

As detailed in Table 9, we have taken a more nuanced approach by identifying specific variations responsible for the degraded performance of each individual model under scrutiny. We have selectively included variations that have a significant impact on a network's performance degradation. Our findings reveal that the most significant impact on the performance of classification models is attributed to Hue variations ranging between –25° and +25°, and by Brightness variations between –10% and +10%, both falling under the category of non-geometric transformations. Additionally, Rotation variations between –45° and +45° and Crop: 0% Minimum Zoom, 20% Maximum Zoom, categorized as geometric transformations, demonstrate a notable effect on the networks' performance.

**Table 9:** Ablation study of all models for specific transformations impacting each model's degraded performance.

**Models**	**Variation**	**Decrease in accuracy**
Vision Transformers	Grayscale	0.05
Vision Transformers	Hue: between –25° and +25°	0.2
ResNet-50	Noise: up to 5% of pixels	0.37
ResNet-50	Hue: between –25° and +25°	0.2
VGG-16	Rotation: between –45° and +45°	0.4
VGG-16	Crop: 0% Minimum Zoom, 20% Maximum Zoom	0.4
DenseNet-121	Hue: between –25° and +25°	0.4
DenseNet-121	Crop: 0% Minimum Zoom, 20% Maximum Zoom	0.45
OpenAI CLIP	Brightness: between –10% and +10%	0.49

#### 4.5.2 Comparison with state-of-the-art methods based on features other than dermoscopic structures

In this subsection, we compared the performance of our technique, which is based on features of dermoscopic structures, with that of techniques relying on features other than dermoscopic structures, such as color, size, and shape of lesions. All these techniques are founded on models comprising CNN architectures or combinations thereof. In the ISIC challenges of 2018 and 2019, numerous teams participated in the melanoma classification task, and the results of their techniques are presented in Table 10. Referring to Table 10, Gessert et al. (2019) utilized a pre-trained model on the ImageNet dataset known as EfficientNets in their technique. They applied scaling rules to make the model adaptable to any image size. Zhou et al. (2019) employed a transfer learning technique by using an ensemble of state-of-the-art deep learning-based models, including se-resnext50, se-resnext101, densenet121, and efficient net-b2, b3, and b4. Pacheco et al. (2019) implemented another ensemble-based approach with CNN models, incorporating SENet, PNASNet, ResNet-50, 101, and 152, InceptionV4, DenseNet-121, 169, and 201, as well as MobileNetV2, GoogleNet, and VGG-16, 19. Sreena and Lijiya (2019) adopted DenseNet-161 for skin lesion classification, fine-tuning the model on a pre-trained ImageNet dataset. Tô et al. (2019) addressed dataset imbalance and overfitting issues by employing EfficientNet and Inception ResNet.

**Table 10:** Performance evaluation results of participants' algorithms in skin lesion classification of ISIC 2018 and 2019.

**Techniques**	**Dataset**	**A**
Ensemble of multi-res efficientNets with SEN154 (Gessert et al., 2019)	ISIC 2019	0.92
Ensemble of EfficienetB3-B4-Seresnext101 (Zhou et al., 2019)	ISIC 2019	0.91
Ensemble classifiers (Pacheco et al., 2019)	ISIC 2019	0.91
Densenet-161 (Sreena and Lijiya, 2019)	ISIC 2019	0.91
CNNs based on inception-resnet, exception net, and EfficientNet (Tô et al., 2019)	ISIC 2019	0.91
Malanet based on DenseNet (Zhang P. et al., 2019)	ISIC 2019	0.89
Class-centroid-based openset ensemble CNNs (Adegun and Viriri, 2021)	ISIC 2019	0.91
Long-tail distribution based classifiers (Adegun and Viriri, 2021)	ISIC 2019	0.91
Softmax ensemble and sigmoid ensemble classifier model (Adegun and Viriri, 2021)	ISIC 2019	0.92
Test time augmentation on ensemble models (Adegun and Viriri, 2021)	ISIC 2019	0.92
Xception, Inception-ResNet-V2, and NasNetLarge (Ahmed et al., 2020)	ISIC 2019	0.92
Top 10 models averaged (Nozdryn-Plotnicki et al., 2018)	ISIC 2018	0.95
Large ensemble with heavy multi-cropping and loss weighting (Gessert et al., 2018)	ISIC 2018	0.97
Emsemble Of SENET and PNANET with Data Augmentation (Li and Shen, 2018)	ISIC 2018	0.96
Densenet (Li and Shen, 2018)	ISIC 2018	0.96
Models average (Amro et al., 2018)	ISIC 2018	0.95
Average of 15 deep learning models (Bissoto et al., 2018)	ISIC 2018	0.95
FV+Res101 (Pan and Xia, 2018)	ISIC 2018	0.95
WonDerM: skin lesion classification with fine-tuned neural networks (Lee et al., 2018)	ISIC 2018	0.95
Resnext101 and DPN92, Snapshot ensamble, D4 TTA (Adegun and Viriri, 2021)	ISIC 2018	0.96
Emsemble Of ResNet-152 (Li and Shen, 2018)	ISIC 2018	0.95
**Our technique [ViTs with convolutions (data augmentation)]**	**DERM7**	**0.98**

The bold selections have been highlighted to showcase our implemented technique/model or to underscore its exceptional accuracy.

Zhang P. et al. (2019) utilized a 169-layer dense attention network backbone to formulate a model named MelaNet. This model's output layers comprised eight units designed for constructing a multi-class classifier. Adegun and Viriri (2021) proposed an ensembling model by employing the mean softmax vector for the probability distribution and training them on the same hyperparameters. Adegun and Viriri (2021) applied a combination of EfficientNets, DenseNet161, and Se-ResNext101, training these models independently. Adegun and Viriri (2021) employed both softmax and sigmoid activation layers for predictions, utilizing seven models with top-1 accuracy. Adegun and Viriri (2021) utilized the Diversity Generator for generating and combining results from diverse ensembling-based models. Ahmed et al. (2020) proposed a fine-tuning-based technique for ensemble-based models, including Xception, Inception-ResNet-V2, and NasNetLarge, trained on 1.2 million images of 1,000 classes. Although the models employed in the ISIC challenge demonstrated strong performance, they proved to be time-consuming and demanded extensive resources for training, making it impractical for fine-tuning numerous models.

In recent approaches, images undergo segmentation as an initial step, followed by feeding these segmented images into improved deep learning-based classification models. According to Table 10, the Large Ensemble method by Gessert et al. (2018) achieved an accuracy score of 97.2%. A notable observation is that the results of ISIC 2018, as presented in the table, exhibit significantly better performance compared to the methods employed in ISIC 2019. It can be deduced from Table 9 that certain ISIC 2018 models display commendable classification accuracy in contrast to their ISIC 2019 counterparts. This improvement is attributed to the positive effects arising from the segmentation of lesion images, a crucial aspect in contemporary classification methods. Prior to their utilization in models for classification, these lesion images undergo preprocessing and segmentation. The results suggest that classifying segmented lesions outperforms the classification of non-segmented lesions. Additionally, the inference can be drawn that less complex models are more suitable for classifying segmented lesion images, given that the models in ISIC 2018 techniques are notably simpler than their ISIC 2019 counterparts.

### 4.6 Significance of using dermoscopic structures as features

In the field of dermatology, slight variations in skin color, size, and shape have historically been crucial factors contributing significantly to the diagnosis of melanocytic lesions. This is evident in dermoscopic examinations where considerable importance has been placed on the color, size, and shape of lesions. However, dermoscopy allows for the visualization of multiple layers of skin structures on the body's surface, such as lines, clods, circles, and dots, providing diagnostic clues independent of color, size, and shape of the lesions. In essence, while it is feasible to categorize melanocytic lesions based on color, they are often more accurately differentiated by their distinctive morphological characteristics and the distribution of specific structures over the lesions. For instance, the characteristic “ovoid nests” of basal cell carcinomas, traditionally perceived as blue-grey, may appear brown under non-polarized dermoscopy, illustrating the significance of morphological features over color.

Supporting this interpretation, our tests on various versions of test sets have revealed no significant differences in accurately diagnosing melanoma in artificially altered images. These tests suggest that melanoma diagnosis can be facilitated by focusing on the characteristics of dermoscopic structures, even when color remains unchanged or is absent.

Compared to a diagnosis based on the color, size, and shape of lesions, a more objective and accurate diagnosis may be achievable by considering dermatologic structures and patterns. Establishing color standards may prove challenging due to the diverse range of dermatoscopes available, each with its unique visual characteristics and light spectrum. Beyond experience, memory, and context, the perception of color, size, and shape is influenced by differences in retinal photoreceptors, as seen in cases of color blindness. In the context of dermatological diagnosis, this suggests that while color, size, and shape may offer utility for diagnostic purposes, they inherently harbor a degree of subjective bias that varies among observers.

Delving into future treatment methodologies, the clinical implications of comprehending dermoscopic patterns will lie in their role in characterizing melanomas based on specific features. This understanding will empower future clinicians to craft personalized treatment plans tailored to the unique characteristics of individual lesions. The practical application will manifest in the realm of automated systems leveraging dermoscopic features. These systems will aid in the stratification of melanomas, providing valuable guidance to clinicians in determining the most appropriate and personalized treatment strategies for their patients.

### 4.7 Testing on ISIC 2016

For evaluation purposes, Vision Transformers were also tested on the ISIC 2016 dataset to determine their classification ability on unseen images, relying on features of dermoscopic structures. This trial-and-error testing provided us with the insight that the models can be employed to infer labels on datasets that lack structural clinical annotations.

## 5 Conclusions

In this paper, we present a scheme for melanoma classification using three different dermoscopic structures with two clinically labeled datasets, namely Ph2, and Derm7pt. Our proposed technique comprises five phases: classification of datasets for training based on dermoscopic structures, obtaining classification results, which include dermoscopic structures, from five models–Vision Transformers, OpenAI Clip, ResNet-50, Vgg-16, and DenseNet-121. We then implement methods to reduce the brittleness of the models and visualize features and, finally, compare the classification results obtained by training the models.

The classification accuracy achieved for various dermoscopic structures by Vision Transformers surpasses that of OpenAI Clip, delivering results between 80–100% for all three dermoscopic structures, as depicted in Table 7. Additionally, the key areas in classifying melanoma lesions are identified as a blue-white veil, dots/globules, and streaks, based on feature visualization that correlates with dermoscopic structures in melanoma detection. Furthermore, research involving dermoscopic structures addresses the susceptibility of AI to image variations. By training Convolutional Neural Networks (CNNs) to differentiate between melanoma and benign lesions, the presence of specific dermoscopic structures remains invariant to natural and artificial changes in images, such as rotation and zooming, thereby significantly enhancing detection accuracy.

However, the number of images in the utilized datasets was limited. Small datasets, like Ph2, may lack the diversity required to represent the full spectrum of dermoscopic variations in melanoma cases. This limitation could hinder the model's ability to generalize to unseen cases. With a small dataset, there's an increased risk of overfitting. Models may memorize patterns specific to the dataset rather than learning generalizable features, resulting in poor performance on new data. Also, small datasets might not adequately represent the variety of skin types, ages, and melanoma types. This can introduce bias and affect the model's ability to generalize to a broader population. Still, we advocate for collecting more clinically annotated datasets based on dermoscopic structures.

In the future, automatic computation of dermoscopic structural features can be achieved by applying deep learning and semantic segmentation, followed by extracting measurements from the segmented results. Another effective approach involves leveraging transfer learning, where pre-trained models on larger datasets, such as ImageNet, are fine-tuned on the smaller melanoma dataset. This allows the model to initialize with knowledge acquired from a more extensive dataset, potentially improving its performance in the specific task of melanoma detection. Additionally, Generative Adversarial Networks (GANs) can be utilized to generate synthetic dermoscopic images. This addresses the lack of diversity in the original dataset by creating realistic images that complement the available data. GANs play a crucial role in broadening the model's exposure to a wider range of melanoma variations. Ensemble methods provide another avenue for enhancement by combining predictions from multiple models. This contributes to overall performance improvement, helping to mitigate overfitting issues and bolstering the generalization capabilities of the melanoma detection model. Exploring biopsy-level analysis is essential for incorporating histopathological information along with dermoscopic features. This integration offers a more comprehensive understanding of melanoma, potentially leading to improved classification accuracy. Lastly, continual learning strategies should be implemented to adapt the model over time as new data becomes available. This dynamic approach ensures that the model remains relevant and effective, evolving alongside the constantly expanding knowledge about melanoma dermoscopic structures.

## Data availability statement

Publicly available datasets were analyzed in this study. This data can be found here: Mendonça et al. (2013) and Kawahara et al. (2018).

## Author contributions

FM: Data curation, Formal analysis, Investigation, Methodology, Software, Validation, Writing—original draft. MY: Conceptualization, Formal analysis, Funding acquisition, Project administration, Resources, Supervision, Visualization, Writing—review & editing. HS: Data curation, Investigation, Supervision, Validation, Visualization, Writing—review & editing. SV: Resources, Supervision, Validation, Visualization, Writing—review & editing.
